# DTNQ-Pro, a Mimetic Dipeptide, Sensitizes Human Colon Cancer Cells to 5-Fluorouracil Treatment

**DOI:** 10.1155/2013/509056

**Published:** 2013-04-21

**Authors:** Isabel Gomez-Monterrey, Pietro Campiglia, Ilaria Scognamiglio, Daniela Vanacore, Alessandra Dicitore, Angela Lombardi, Michele Caraglia, Ettore Novellino, Paola Stiuso

**Affiliations:** ^1^Department of Pharmaceutical and Toxicological Chemistry, University of Naples Federico II, Naples, Italy; ^2^Department of Pharmaceutical Sciences, University of Salerno, Fisciano, Salerno, Italy; ^3^Department of Biochemistry, Biophysics and General Pathology, Second University of Naples, Naples, Italy; ^4^Italian Institute for Auxology, IRCC, 20145 Milan, Italy

## Abstract

The resistance of growing human colon cancer cells to chemotherapy agents has been correlated to endogenous overexpression of stress proteins including the family of heat shock proteins (HSPs). Previously, we have demonstrated that a quinone-based mimetic dipeptide, named DTNQ-Pro, induced differentiation of growing Caco-2 cells through inhibition of HSP70 and HSP90. In addition, our product induced a HSP27 and vimentin intracellular redistribution. In the present study, we have evaluated whether a decrease of stress proteins induced by DTNQ-Pro in Caco-2 cells could sensitize these cells to treatment with 5-fluorouracil (5-FU) cytotoxicity. The pretreatment of Caco-2 with 500 nM of DTNQ-Pro increases lipid peroxidation and decreases expression of p38 mitogen-activated protein kinase (MAPK) and FOXO3a. At the same experimental conditions, an increase of the 5-FU-induced growth inhibition of Caco-2 cells was recorded. These effects could be due to enhanced DTNQ-Pro-induced membrane lipid peroxidation that, in turn, causes the sensitization of cancer cells to the cytotoxicity mediated by 5-FU.

## 1. Introduction

Adenocarcinoma cells, such as colorectal cancer (CRC) cells, are remarkably resistant to radiation or chemotherapy-induced damage. As a consequence, the tumours are hard to treat and often proliferate rapidly, even under conditions that may adversely affect normal cells. For several years, 5-fluorouracil (5-FU), a pyrimidine antimetabolite, has been the drug of choice for the treatment of CRC as well as head and neck, pancreatic, and breast carcinomas. 5-FU is known to block DNA synthesis by the inhibition of thymidylate synthase (TS), which is regulated by cell cycle proteins controlled by phosphorylation [1]. Unfortunately, many of the schedules based upon 5-FU alone or in combination with other agents become ineffective during the course of the treatment due to the occurrence of drug resistance to 5-FU. Between several survival pathways activated in cancer cells to antagonize the antiproliferative activities of antineoplastic agents [2–4]. The mechanisms underlying the survival advantage can also be partially related to the increased expression of stress proteins [5, 6]. In fact, in contrast to normal cells, the basal levels of inducible heat shock proteins (HSPs) are frequently higher in tumour cells [7, 8]. The high expression of members of the HSP family in CRC cells has been associated with both metastases and resistance to chemotherapy. Moreover, in experimental models, HSP27 and HSP70 have been shown to increase tumorigenicity of cancer cells, and HSP depletion can induce a spontaneous regression of the tumour [9–11]. Recent data provide direct evidence on the association between HSP27 protein expression levels and 5-FU sensitivity in Caco-2 cells. In fact, the suppression of HSP27 expression in these cells may promote 5-FU sensitivity by inducing apoptosis, despite the acceleration in 5-FU metabolism [12]. 

HSP27 has been also implicated in a wide range of cell functions including cell protection, differentiation, and cell proliferation [13–15]. Moreover, HSPs are often associated with specific lipids or particular membrane areas (such as lipid rafts). In this light, changes of membrane physical state alter HSP gene expression [16–18]. The correlation between HSPs and control of cell proliferation is demonstrated by the fact that they are targets of important cell growth regulators such as mitogen-activated protein kinases (MAPKs) [19]. Moreover, the latter can be modulated by HSP multichaperone complex that protects MAPKs from proteasome-mediated degradation [20]. In addition, it has been reported that the inhibition of the multichaperone complex can sensitize cancer cells to agents raised against ERK-mediated pathways [21].

In mammalian cells, there are three well-characterized subfamilies of MAPKs: the extracellular signal-regulated kinases (ERK), the c-Jun N-terminal kinases (JNK, also known as the stress-activated protein kinases), and the p38 MAPK kinases. Each MAPK is activated through a specific phosphorylation cascade. ERK pathway is activated in response to growth factors, conferring a survival advantage on cells [22]. In contrast, JNK and p38 MAPK are activated in response to a variety of environmental stressors and inflammatory cytokines, which are frequently associated with the induction of apoptosis [23, 24]. Moreover, it was demonstrated that the p38 MAPK-Hsp27 axis plays an essential role in cancer stem cells-mediated Cis-platinum resistance in oral cancer [25]. Therefore, the development of specific modulators of HSP expression could be desirable either to shed further light on the functional roles of these important proteins in cell survival and in cell death or to further develop new clinically useful antitumour drugs.

In our previous report [26], we have demonstrated that DTNQ-Pro, a quinone-based pentacyclic derivative, modulated cellular redox status inducing cell cycle arrest and differentiation and, finally, driving cells to programmed cell death. Moreover, after 48 h of DTNQ-Pro treatment a decrease of mitochondrial superoxide anion induced by manganese superoxide dismutase (MnSOD) was recorded in Caco-2 cells. This effect was followed by a subsequent decrease of HSP70 expression and an increased membrane lipid peroxidation. The oxidative damage of the membrane induced a redistribution of both HSP27 and vimentin and forced Caco-2 cells to differentiate into enterocytes. On the other hand, the second treatment with DTNQ-Pro activated the apoptotic pathway. The ability of DTNQ-Pro to shift the undifferentiated Caco-2 cells to differentiated enterocytes and then undergo a process of programmed cell death strongly suggests that this compound should be additionally investigated for its potential use in new combination chemotherapy for colon cancer. The goal of the present study was to determine if the pretreatment with DTNQ-Pro can modulate the cytotoxic response of Caco2 cells to fluorouracil (5-FU) and assess the potential mechanisms underlying such modulation. In this report, we showed that increased Caco-2 cell differentiation by pretreatment with DTNQ-Pro enhanced the cytotoxic response of human colon carcinoma cells to 5-FU by modulating the expression of molecules that are associated with either drug sensitivity or resistance. 

## 2. Material and Methods

### 2.1. Cell Cultures

Human colon adenocarcinoma Caco-2 cells (American Type Culture Collection, Rockville, MD, USA) were grown at 37°C in h-glucose MEM containing: 1% (by vol) nonessential amino acids and supplemented with 10% (by vol) decomplemented fetal bovine serum (FBS) (Flow, McLean, VA, USA), 100 U·mL^−1^ penicillin, 100 mg·mL^−1^ streptomycin, 1% L-glutamine, and 1% sodium pyruvate. Cells were grown in six multiwell plates (17–21 passages) in a humidified atmosphere of 95% air/5% CO_2_ at 37°C. After incubation for 4 h in Dulbecco's modified Eagle's medium (DMEM) with 10% FBS, the cells were washed with 1% phosphate-buffered saline (PBS) to remove unattached dead cells and were incubated with 500 nM concentration of DTNQ-Pro for 48 h (D-Caco-2). All experiments were performed in triplicate.

### 2.2. Evaluation of Growth Inhibition of D-Caco-2 Cells

We assessed the sensitivity of the to 5-FU using a microplate colorimetric assay that measures the ability of viable cells to transform a soluble tetrazolium salt (MTT) [27] to an insoluble purple formazan precipitate. D-Caco-2 cells were plated at the appropriate density in 96-well microtitre plates. After 4 h, cells were exposed to different concentration (0–200 *μ*M) of 5-FU for 48 h. 50 mL of MTT (1 mg·mL^−1^) and 200 mL of medium were added to the cells in each well. After a 4 h incubation at 37°C, the medium was removed; then the formazan crystals were solubilized by adding 150 mL of DMSO and by mixing it in an orbital shaker for 5 min. Absorbance at 550 nm was measured using a plate reader. Experiments were performed in triplicate. The percent inhibition of the treated cells was calculated by the following formula:

(1) $$\begin{array}{c} \%\;\;\text{Inhibition}=\frac{{\text{A}}_{550}\;\text{treated}}{{\text{A}}_{550}\;\text{CTR}}. \end{array}$$

#### 2.2.1. Alkaline Phosphatase (ALP) Activity Evaluation

ALP activity was used as marker of the degree of differentiation of D-Caco-2 cells. Attached and floating cells were washed and lysed with 0.25% sodium deoxycholate, essentially as described by Herz et al. [28]. ALP activity was determined using Sigma Diagnostics ALP reagent (no. 245). Total cellular protein content of the samples was determined in a microassay procedure as described by Bradford [29] using the Coomassie protein assay reagent kit (Pierce). ALP activity was calculated as units of activity per milligram of protein.

### 2.3. Lipid Peroxidation Assay

Lipid peroxidation was evaluated using an analytical quantitative methodology. It relies upon the formation of a coloured adduct produced by the stechiometric reaction of aldehydes with thiobarbituric acid (TBA). The thiobarbituric acid reactive substances (TBARS) assay was performed on membranes extracted from cells, using an icecold lysis buffer (50 mM Tris, 150 mM NaCl, 10 mM EDTA, 1% Triton) supplemented with a mixture of protease inhibitors. The homogenate was centrifuged at 1200 g for 10 min in order to separate cytosol (supernatants) from membranes (pellet). The pellet was dissolved in 50 mM Tris, 150 mM NaCl, and 10 mM EDTA, and the protein content of the samples was determined by Bio-Rad assay (Bio-Rad Laboratories, San Diego, CA, USA). Aliquots (10 mL) of the membrane preparation were added to 2 mL of TBA-trichloroacetic acid (TCA) (15% TCA, 0.3% TBA in 0.12 N HCl) solution at 100°C for 30 min. The reaction was stopped by cooling the sample in cold water, and, after a centrifugation at 15 000 g for 10 min, the chromogen (TBARS) was quantified by spectrophotometry at a wavelength of 532 nm. The amount of TBARS was expressed as mM·mg^−1^ proteins. All data are the mean ± SD of three experiments.

### 2.4. Western Blot Assay

The effects of DTNQ-Pro on expression of HSP90, p38, p38, and FOXO3a were determined by Western blots. The cells lysates were prepared using an ice-cold lysis buffer (50 mM Tris, 150 mM NaCl, 10 mM EDTA, 1% Triton) supplemented with a mixture of protease inhibitors containing antipain, bestatin, chymostatin, leupeptin, pepstatin, phosphoramidon, pefabloc, EDTA, and aprotinin (Boehringer, Mannheim, Germany). Equivalent protein samples were resolved on 8%–12% sodium dodecyl sulphate-polyacrylamide gels and transferred to nitrocellulose membranes (Bio-Rad, Germany). For immunodetection, membranes were incubated overnight with specific antibodies at the concentrations recommended by the manufacturer. All antibodies were diluted in Tris buffered saline/Tween 20–1% milk powder. This step was followed by incubation with the corresponding horseradish peroxidase conjugated antibody (anti-mouse IgG 1 : 2000, anti-rabbit IgG 1 : 6000, Biosource, Germany). Bands were analysed by enhanced chemiluminescence (ECL kit, Amersham, Germany).

## 3. Results

### 3.1. Cytotoxic Effects Induced by 5-FU on Growing D-Caco-2 Cells

**Figure 1:**
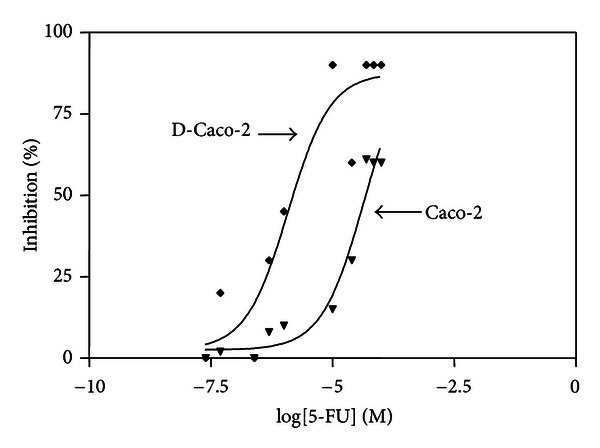
Sensitivity of the D-Caco-2 cells the to 5-FU. Both Caco-2 and D-Caco-2 cells were plated in 96-well microtitre plates at the density of 3 × 10^3^ cells/plate. After 4 h, cells were exposed to different concentration (0–200 *μ*M) of 5-FU for 48 h. The percentage of inhibition was calculated with the formula reported in methods. Experiments were performed in triplicate.

Previously, we have shown the biochemical events elicited by DTNQ-Pro, a mimetic peptide, in growing human colon adenocarcinoma cells. Undifferentiated Caco-2 treated for 48 h with 500 nM DTNQ-Pro presented an increased membrane lipid peroxidation and a redistribution of both HSP27 and vimentin [26]. Moreover, growing Caco-2 cells differentiate into enterocytes. Here the effect of conventional cytotoxic agent 5-FU was studied on exponentially growing pretreated DTNQ-Pro Caco-2 cell lines (D-Caco-2). Cells were treated for 48 h with 500 nM DTNQ-Pro and, thereafter, different concentrations of 5-FU (0–200 *μ*M) were added to the cells for 48 h. In exponentially growing cells, pretreatment with DTNQ-Pro potentiated the cell growth inhibition observed with 5-FU alone (Figure 1). As shown in Table 1, for growing Caco-2 cells, the concentration of 5-FU (46 *μ*M) that induced 50% of growth inhibition (IC:50) was reduced about 30-fold in D-Caco-2 (1.2 *μ*M). All successive experiments were performed on Caco-2 cell, treated for 48 h with 500 nM DTNQ-Pro (D-Caco-2).

### 3.2. 5-FU Increases Cell Cycle Arrest and D-Caco-2 Differentiation

**Figure 2:**
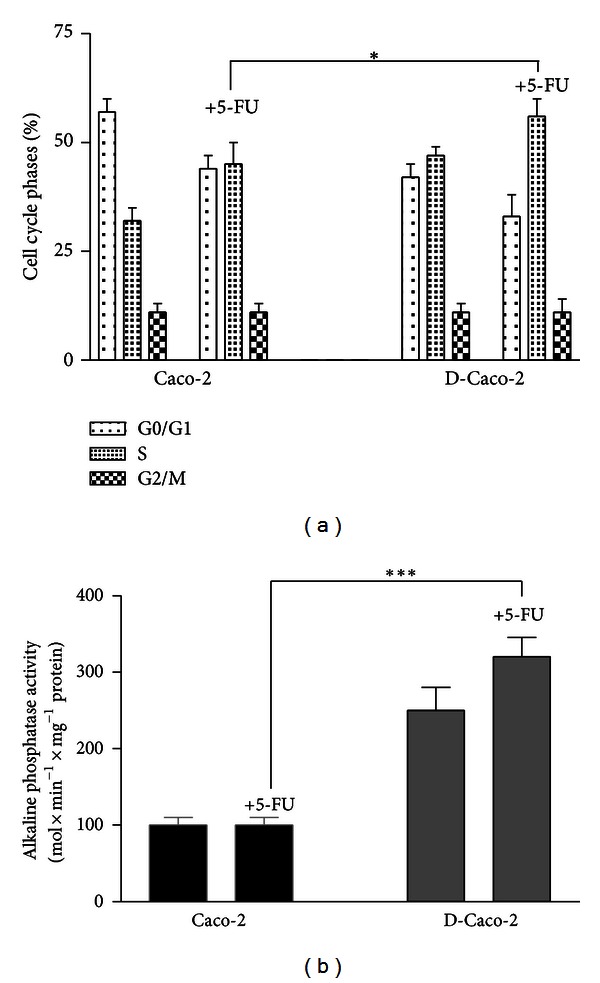
(a) Effects of 5-FU on the distribution of Caco-2 and D-Caco-2 cells populations. Data represent the percentage of cells in each phase of the cell cycle. Cell cycle distribution was determined by DNA flow cytometric analysis. Samples from preconfluent Caco-2 and D-Caco2 cells was analysed after 48 h of treatment with 500 nM 5-FU. Numbers indicate percentage of cells in G0/G1, S and G2/M phases. Data are representative of four separate analyses. (b) Differentiation effects of 5-FU of Caco-2 and D-Caco-2. The differentiation was assessed by measurement of ALP activity after 48 h of culture with 500 nM of 5-FU. Summary data shown are means ± SEM (*n* = 4; ***P < 0.001).

**Figure 3:**
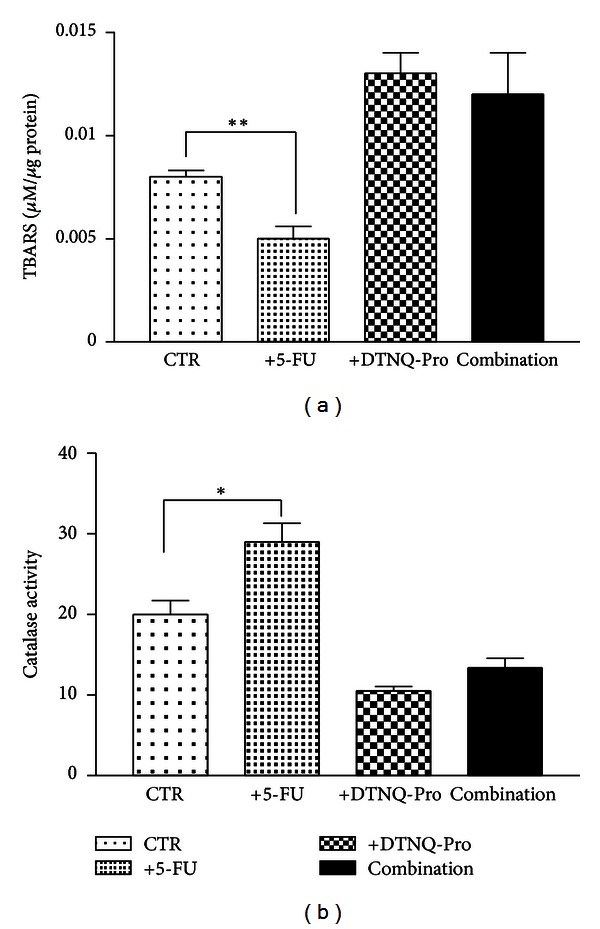
Effect of 5-FU D-Caco-2 treatment on TBARS. The cells were seeded in six multiwell plates at the density of 25 × 10^4^ cells/plate and were incubated for the first 48 h with 500 nM DTNQ-Pro (D-Caco-2) the cells were washed to remove unattached dead cells and were incubated with and without 500 nM of 5-FU for subsequently 48 h. (a) TBARS levels in Caco-2 (CTR) and D-Caco-2 cells after 48 h of incubation without and with 500 nM 5-FU; (b) catalase activity. Catalase activity in Caco-2 (CTR) and D-Caco-2 cells after 48 h of incubation without and with 500 nM 5-FU. The bars represent means ± SEM of three independent experiments. Asterisks indicate significant difference between the D-Caco-2-treated samples compared with control value **P < 0.003; *P < 0.05; n.s.: not significant.

5-FU is an antimetabolite known to specifically block cells in S phase. To elucidate whether 5-FU treatment of D-Caco-2 determined cell cycle perturbation, we analysed the percentage cell cycle by FACS analysis. Cell cycle analysis of Caco-2 and D-Caco-2 cells revealed a percentage of cells of 54 ± 4 and 42 ± 6% in G0/G1 phase, 35 ± 5 and 47 ± 5% in S phase, and 11 ± 1% and 11 ± 2% in G2/M phase, respectively (Figure 2). The additional treatment of the cells with 500 nM 5-FU showed a significant (*P* < 0.014) accumulation of D-Caco-2 cells (56%) in S phase if compared to Caco-2 (45%) and a concomitant G0/G1 phase decrease (33%) if compared to Caco-2 cells (44%). As cell division arrest is one of the biological effects required for cell differentiation [30], we determined the effect of DTNQ-Pro on D-Caco-2 differentiation. In Figure 3(b) it is shown that ALP activity, as a marker of differentiation into enterocytes, correlated to postconfluent phase [31]. ALP activity was about 3-fold increased (*P* < 0.001) only in D-Caco-2 cells treated with 500 nM 5-FU while it remained unaltered in the other cases.

### 3.3. Lipid Peroxidation and Catalase Activity in D-Caco-2 Cells after 5-FU Treatment

In Figure 3 we reported the values of both TBARS, as lipid peroxidation markers [32], and catalase activity, as scavenger enzyme, in both Caco-2 and D-Caco2 cells treated with 500 nM 5-FU. The incubation of Caco-2 cells with 5-FU determined a statistically significant decrease of TBARS values (*P* < 0.0015) with a concomitant increase of the catalase activity (*P* < 0.0124). In the D-Caco-2 the basal levels of TBARS and catalase activity were 1.6-fold and 0.5-fold higher, respectively, than those recorded in Caco-2 cells. No changes were observed in both TBARS values and catalase activity when the D-Caco-2 cells were incubated with 500 nM of 5-FU.

### 3.4. Evaluation of the Expression of Molecular Factors Involved in the Tumour Cell Resistance to 5-FU

**Figure 4:**
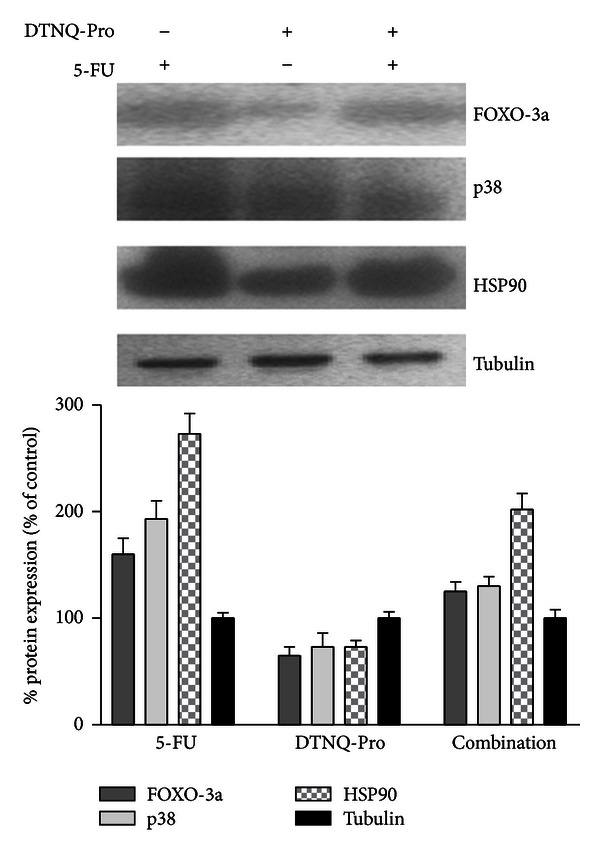
Expression of Foxo3a, p38, and HSP90 in Caco-2 and D-Caco-2 cells treated for 48 h with 5-FU. The cells were incubated with 500 nM of 5-FU, and the protein expression was evaluated by Western blotting. All the experiments were performed at least three times with similar results. The graphs show the summary data (as % of expression in untreated cells), normalized to *γ*-tubulin expression after 48 h of treatment with 5-FU. Data shown are means ± SEM (*n* = 4; *P < 0.05, **P < 0.003).

We have evaluated, in D-Caco2 cells, the effects of 5-FU treatment on the expression of FOXO-3a, HSP90 and p38 MAPK proteins that are involved in mechanisms of cell resistance to 5-FU. Western blot and densitometric analysis of HSP90, FOXO-3a, and p38 MAPK in Caco2 after DTNQ-Pro treatment is shown in Figure 4. The Caco-2 cells expressed high levels of Hsp90, and this expression was 0.5-fold lower in D-Caco2. Furthermore DTNQ-Pro Caco-2 treatment induced about 0.5-fold decrease of both p38 and FOXO-3a expression. On the other hand, 5-FU induced about 3- and 2-fold increase of Hsp90 levels in Caco-2 and D-Caco-2 cells, respectively; moreover 5-FU weakly increased the expression of FOXO-3a and p38 in D-Caco-2 cells. 

## 4. Discussion

Previously, we showed that DTNQ-Pro acts on two major targets involved in the resistance of CRC cells to chemotherapy agents: the process of cell differentiation and overexpression of HSP family proteins [26]. We have demonstrated that DTNQ-Pro induced cell membrane modifications by redistribution of HSP27 and vimentin within the cell. Moreover, DTNQ-Pro inhibited the growth by inducing S phase cell cycle arrest and increased cellular differentiation. 

In the present study, we show that DTNQ-Pro, a mimetic dipeptide, sensitizes human CRC cells to the conventional chemotherapy agent 5-FU. Exposure of Caco-2 cells to DTNQ-Pro induced a hydrogen peroxide (H_2_O_2_) increase due to decreased catalase activity. At the same time, an increased rate of lipid peroxidation was recorded as evaluated by TBARS production. Lipid peroxidation occurred together with cell differentiation as demonstrated by the increased ALP activity. The treatment of D-Caco-2 cells with 5-FU caused an additional increase of both ALP activity and cell accumulation in S phase, while no increase of catalase activity or antagonism on TBARS values was recorded. Pretreatment of growing Caco-2 cell lines with 500 nM of DTNQ-pro (D-Caco2) induced also a decrease of FOXO3a protein expression with a consequent downregulation of D-cyclin (data not shown) and an accumulation of Caco-2 cells in the S-phase of the cell cycle. In fact, FOXO transcription factors [33] regulate the expression of antioxidants enzymes such as SOD and Catalase but, in addition to regulation of antioxidants, Foxo is also involved in the transcriptional upregulation of cell cycle inhibitors, including p21, p27, and p130, and downregulation of D-type cyclins. Therefore, our data demonstrate that S phase accumulation occurred together with regulation of transcriptional factors that are involved in the regulation of cell cycle.

5-FU is known to block DNA synthesis by the inhibition of thymidylate synthase (TS), which is regulated by cell cycle proteins controlled by phosphorylation [1]. Increased TS expression in tumors is an underlying mechanism by which tumor cells can escape from the toxic effect of 5-FU and become drug resistant [34]. In fact, the increased expression of the target is a generalized mechanism of resistance of tumour cells to antitumour agents determining an increase of the drug concentration needed to inhibit the molecular target. 

DTNQ-Pro induces both cell accumulation in S phase and differentiation, with a probable decrease in the *de novo* DNA synthesis. On the basis of these considerations, we hypothesize that the level of TS expression could be reduced and this reduction may be directly responsible for the increase in sensitivity to 5-FU. Finally, we have found that DTNQ-Pro reduced HSP90 and p38 MAPK proteins. Hsp90 is a ubiquitous cellular protein and its function as a molecular chaperone is vital for cell survival [35] while p38 kinase is a final mediator of stress-induced pathways. We hypothesize that the increased responsiveness to 5-FU of D-Caco-2 could be attributed to (i) block of cell cycle in S phase and differentiation, (ii) decrease of HSP90 and p38 MAPK protein that can, in turn, promote the antiproliferative effects of 5-FU, and (iii) decreased expression of the 5-FU target. Our data provide evidence that DTNQ-pro is a promising chemotherapeutic agent that increases the chemosensitivity of growing adenocarcinoma colon cancer cells to 5-FU treatment.

## Figures and Tables

**Table 1:** IC50 5-FU values (micromolar) in growing human colon carcinoma cell lines with or without DTNQ-Pro pretreatment. Values represent means ± SEM (*n* = 8), *P* < 0.05.

	IC:50 on Caco-2	IC:50 on D-Caco-2
5-FU	46 *μ*M	1.2 *μ*M
